# A Rare Presentation of Retiform Hemangioendothelioma in the External Auditory Canal

**DOI:** 10.1155/2014/715035

**Published:** 2014-07-13

**Authors:** Ezhil Arasan Jothi, Muthuchitra Sundaram, Jeyalakshmidevi Namasivayam, Mathivanan Jothi

**Affiliations:** ^1^Department of Otorhinolaryngology, Government Thiruvarur Medical College Hospital, Thiruvarur, Tamil Nadu 610004, India; ^2^Department of Pathology, Government Thiruvarur Medical College Hospital, Thiruvarur, Tamil Nadu 610004, India; ^3^Department of Pathology, Government Villupuram Medical College, Villupuram, Tamil Nadu 605601, India; ^4^Dulbecco Telethon Institute and Division of Regenerative Medicine, San Raffaele Scientific Institute, 20132 Milan, Italy

## Abstract

Retiform hemangioendothelioma is a rare intermediate or borderline neoplasm of the blood vessels that mostly occurs in extremities. Here we report a unique case of retiform hemangioendothelioma presented in the external auditory canal. 58-year-old male patient presented with the complaint of right ear swelling for 4 years. On examination, a spherical swelling in the right ear was found occluding the view of external auditory canal. The tumor was removed surgically. Intraoperatively, the mass was found attached to the outer part of the right external auditory canal near the root of helix. Histopathology of the resected tumor showed typical features of retiform hemangioendothelioma. In addition, immunohistochemical analysis revealed that tumor was positive for endothelial cell marker CD34 and occasionally positive for cell proliferative marker Ki-67.

## 1. Introduction

Retiform hemangioendothelioma (RH) is a rare distinctive variant of low-grade cutaneous angiosarcoma typically present on the extremities [1–4]. Till now, less than 35 cases were reported worldwide which include two cases of canine retiform hemangioendothelioma [5–8]. RH tumor is first described in 1994 and it is closely related to Dabska tumor (DT) [2]. The term retiform hemangioendothelioma was coined due to its vascular channel resemblance with the rete testis (retiform) and its borderline malignant behavior (hemangioendothelioma) in contrast to the benign angioma and the malignant angiosarcoma [2, 6, 9, 10]. These tumors occur in the patients on their second to fourth decades of life with the mean age of 36 years [2, 11]. The tumor has predilection towards females with the ratio of 2 : 1 [9, 11]. RH may present as a slow growing mass, plaque-like lesion, or dermal or subcutaneous nodule and it usually develops as a single solitary lesion; however, multiple lesions were also reported elsewhere [2, 7, 12]. Retiform hemangioendothelioma has a high local recurrence rate and it rarely metastasizes [2, 6, 13]. Till date, only one case of RH related death has been reported [4]. A typical microscopic feature of RH is the presence of elongated, arborizing, thin-walled blood vessels ramifying through the dermis in a retiform pattern which is reminiscent of the normal rete testis architecture [2, 11]. The blood vessels in RH are lined by hobnail endothelial cells with focal intraluminal papillary projections [2, 14]. Surgery is the most effective form of treatment and unresectable tumors are treated with chemo- and radio-therapies [7, 14].

## 2. Case Report

**Figure 1:**
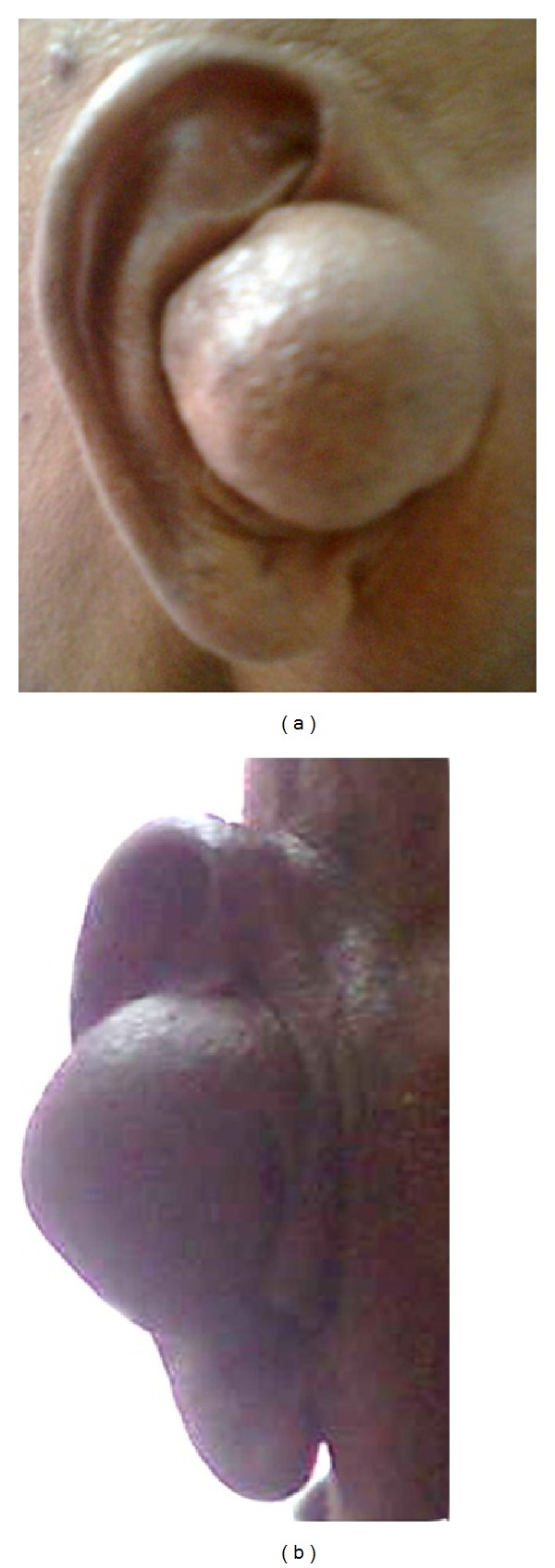
Preoperative photograph of the patient's lateral view shows a spherical shaped swelling in the right ear obscuring the view of external auditory canal (a) and anteroposterior view shows that his tragus has been pushed anteroinferiorly by the tumor mass (b).

**Figure 2:**
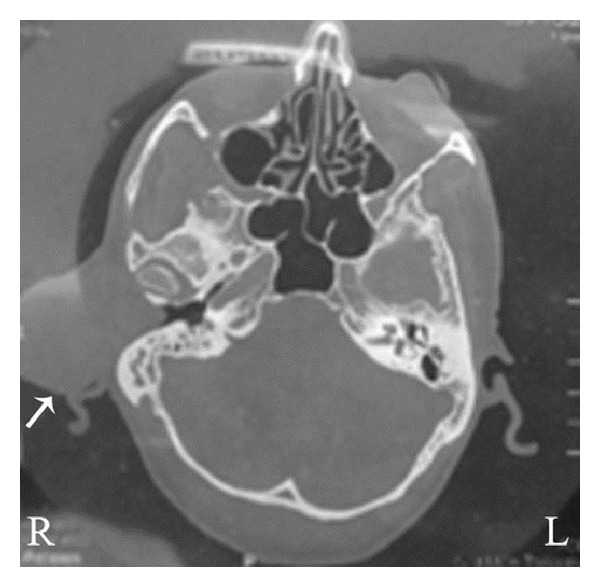
CT image shows the axial section of both temporal bones. The arrow indicates the tumor mass situated anterior to the right pinna involving outer part of the right external auditory canal.

**Figure 3:**
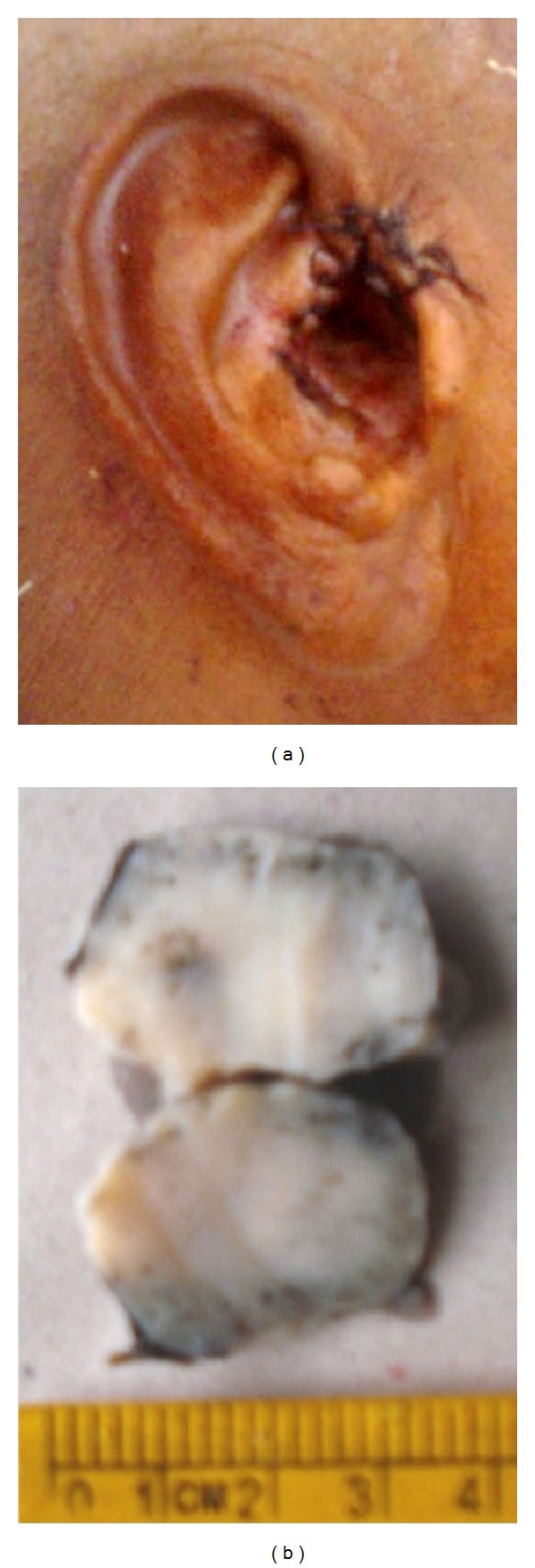
(a) The postoperative photograph shows that the tragus has been repositioned in its original position after excising the tumor. Sutured area in the same image shows the mass attached to the outer part of the right external auditory canal. (b) Photograph of the excised mass showing grey-white and grey-brown area in its cross section.

A 58-year-old male patient visited our outpatient department with the complaint of swelling in the right ear for the past 4 years. He reported that he did not feel any pain or difficulty in hearing. There was no relevant family history. He did not undergo any kind of treatment till his day of visit. On preliminary examination, we have found a large spherical shaped swelling with the size of 4 × 4 cm in the right ear obscuring the view of external auditory canal (Figure 1(a)). The swelling was firm in consistency with areas of fluctuations with mild tenderness. Tragus had been displaced anteroinferiorly due to the swelling (Figure 1(b)). He did not have any significant cervical lymph nodes. Computed tomographic scan (CT scan) of temporal bones showed a heterogeneous mass which was attached to the anterior part of the right pinna near the preauricular region and also suggested that the mass could be a dermoid cyst (Figure 2). We performed fine needle aspiration cytology (FNAC) and the results showed presence of only hemorrhagic smear; subsequently it was reported as a probable cystic lesion with inflammatory pathology. The mass was resected surgically. Intraoperatively, we have found the mass attached to the anterior end and the roof of the right external auditory canal near the root of helix and to the medial surface of the tragus and the tumor was also attached to the overlying skin. After tumor removal, the displaced tragus was secured in its original place and the canal was packed with medicated gauze to prevent stenosis of the ear canal (Figure 3(a)). The resected mass had a mixture of grey-white and brown areas in its cross section (Figure 3(b)).

## 3. Histopathological Features

**Figure 4:**
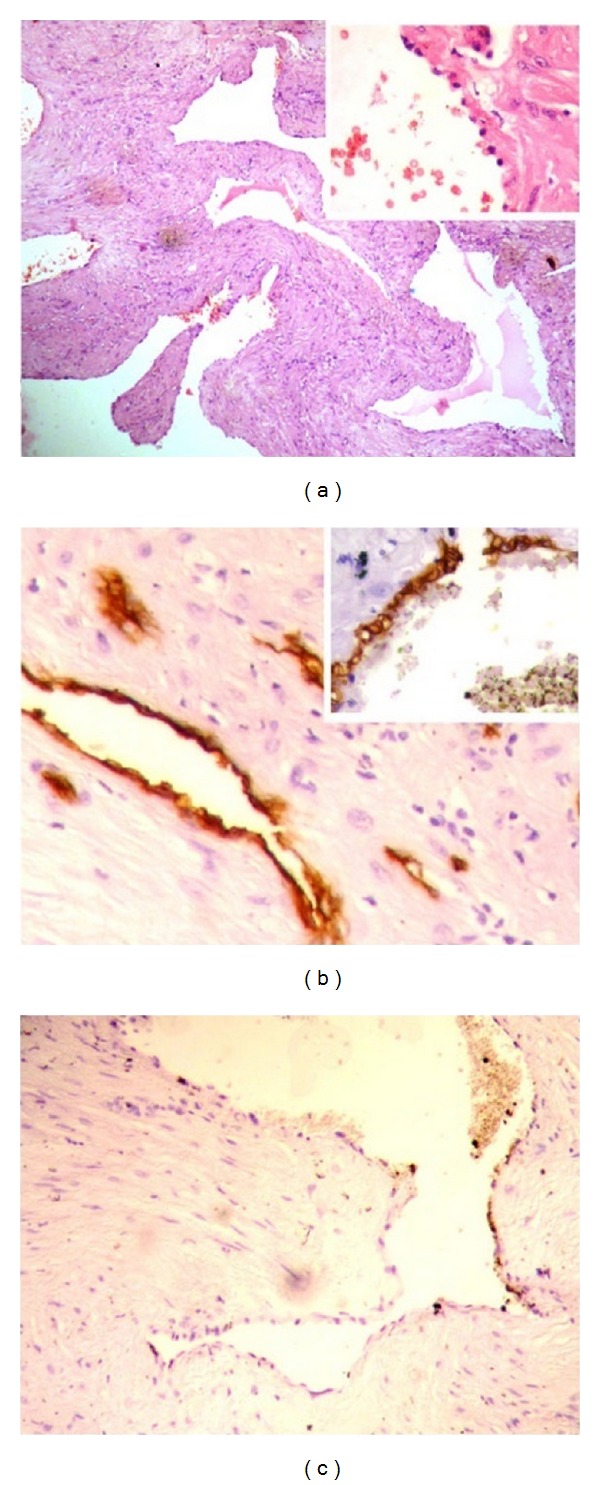
(a) Histopathological image of the tumor shows that the vascularity of the tumor and the blood vessels are arranged in retiform pattern (H&E staining, magnification ×10). The same section under high magnification shows classical hobnail type of endothelial cells projecting into the lumen of the vascular channels (magnification ×40, window). (b) Immunohistochemical staining of sectioned tumor indicates that the tumor cells are positive for CD34 (magnification ×10, ×40 (top right window)) and occasionally positive stain for Ki-67 (c).

Histopathological examination of the resected tumor showed epidermis with a well-demarcated neoplasm in the dermis composed of numerous elongated, tortuous, intercommunicating blood vessels; a few appeared ectatic and some with papillary infoldings (Figure 4(a)). Many are lined by hobnail type of endothelial cells (Figure 4(a), top right window). The vessels are surrounded by collagenous stroma with prominent lymphocytic infiltrate (Figure 4(a)). The tumor is seen infiltrating into the underlying reticular dermis and subcutis. No cytological atypia or mitotic figures are noted in H&E staining. These tumor sections were analyzed immunohistochemically. The immunohistochemical analysis showed that the tumor cells reacted with endothelial marker CD34, which is usually expressed by normal and abnormal endothelial cells (Figure 4(b)). The positive stain for cell proliferative marker Ki-67 was observed only in few areas of the tumor sections (Figure 4(c)).

## 4. Discussion

Retiform hemangioendothelioma is a rare intermediate or borderline malignancy of the blood vessels [1, 11, 13]. It has frequent recurrence rates with rare systemic metastasis [2]. This tumor occurs in second to fourth decade of life with the mean age of 36 years [2, 6, 11]. However, RH is also reported in patients belonging to less than 10 years of age [15]. In our case, the age of the patient is 58 years at the time of diagnosis. The male to female ratio is significantly skewed towards female predominance [9, 11]. It is reported that it may present as a slow growing mass, plaque-like lesion, or dermal or subcutaneous nodule [2, 12]. It usually develops as a solitary lesion, but multiple lesions also are reported [2, 12]. Our patient had a solitary lesion in the ear and we did not notice any other lesions in the body. It mostly occurs in the limbs especially in lower limbs and it can also occur in other parts like trunk, head, penis, and so forth [12, 16, 17]. Most of the cancers in the ear canal are squamous cell carcinomas and other types of cancers like melanoma and adenocarcinoma were also reported [18–20]. O'Duffy and coworkers reported a case of RH involved in the pinna of a 18-year-old man [8]. Zhang and colleagues reported a RH case with plaque-like lesion over the scalp and they have also reported another lesion behind the right ear in the postauricular region [4]. Apart from these there is no other report that shows the involvement of RH in the ear. We have reported a unique case of RH presented in the external auditory canal. Specifically, in our case the tumor was found attached to the anterior part and the roof of the right external auditory canal near the root of helix (Figures 1(a) and 1(b)). Histopathology of the excised mass revealed the tumor blood vessels arborized in retiform pattern which resembled the architecture of normal rete testis (Figure 4(a)). Immunohistochemically RH cells were reported to react with CD31, CD34, factor VIII related antigen, and ulex europaeus agglutinin [2, 7]. In our case, the tumor cells reacted with the endothelial cell marker CD34 (Figure 4(b)). These tumor cells were occasionally positive for cell proliferative marker Ki-67 suggesting the low proliferative potential of RH tumor (Figure 4(c)). Collectively, these results from immunohistochemistry suggested that RH tumors have low mitotic activity and are less aggressive in nature.

The retiform hemangioendothelioma can be differentiated from the closely related diseases like Dabska tumor, well-differentiated angiosarcoma, and hobnail hemangioma by its histological appearance and morphological features. Dabska tumor shows blood vessels with numerous papillary tufts, with a central hyaline core lined by hobnail-like endothelial cells protruding into the lumina [13], whereas retiform hemangioendothelioma has a few papillary infoldings instead of papillary tufts and the retiform type of blood vessels is only seen in RH tumor but not in DT (Figure 4(a)) [6, 13]. Hobnail hemangioma differs from RH by presenting as a small solitary lesion, consisting of a brown to violaceous papule surrounded by a thin pale area and a peripheral ecchymotic ring; microscopically, these tumors are situated more superficially, lack a retiform architecture, and have hobnail endothelial cells that are mainly seen in the vessels near the surface [11]. Similarly, well-differentiated angiosarcoma having nuclear atypia and mitotic figures in cytology and the vessels do not have retiform appearance and the neoplastic channels in angiosarcoma are irregular in shape, intercommunicate with one another in a sinusoidal fashion, and infiltrate surrounding tissues in a destructive pattern and also angiosarcoma has higher incidence of recurrence and metastasis and tumor related mortality than retiform hemangioendothelioma [14].

The treatment of choice for RH would be surgical excision with histopathologically proven tumor-free margins. Half of the RH tumors have local or regional nodal recurrence and these tumors can be successfully treated with radiation therapy. We have treated our patient surgically, and the patient was under regular follow up for the past 16 months. During this period our patient does not show any recurrence from the site of surgery or any new swelling or mass from other parts of the body.

## 5. Conclusion

RH tumors are rare and of low-grade angiosarcoma of the skin, so far less than 35 cases were reported worldwide. The case reported here shows typical features of retiform hemangioendothelioma. RH tumors usually occur in extremities and rarely in other sites. However, according to our knowledge, the case we have reported here is the unique one having a tumor in right external auditory canal. Thus, the presentation of rare cases like this will expand the current knowledge about this rare type of tumor and its sites of involvement which will be of immense help for future references.
